# Editorial: The role of tumor microenvironment in primary liver cancer therapeutic resistance

**DOI:** 10.3389/fonc.2022.938557

**Published:** 2022-07-27

**Authors:** Zhangya Pu, Zhigang Ren, Qiuran Xu, Xiaochen Wang, Jian Chen, Jiang Chen

**Affiliations:** ^1^ Department of Infectious Disease, The First Affiliated Hospital, School of Medicine, Zhejiang University, Hangzhou, China; ^2^ Department of Infectious Diseases, The First Affiliated Hospital of Zhengzhou University, Zhengzhou, China; ^3^ Laboratory of Molecular Diagnosis, Zhejiang Provincial People’s Hospital and Affiliated People’s Hospital, Hangzhou Medical College, Hangzhou, China; ^4^ Department of Immunology, University of Texas Southwestern Medical Center, Dallas, TX, United States; ^5^ Department of Research and Development, BioAtla, Inc., San Diego, CA, United States; ^6^ Department of General Surgery, Sir Run Run Shaw Hospital, Zhejiang University, Hangzhou, China

**Keywords:** hepatocellular carcinoma, tumor microenvironment, immune infiltration, biomarkers, prognostic model

Hepatocellular carcinoma (HCC) is the most prevalent histological type of primary liver cancer, ranking as the sixth most common malignancy and the third leading cause of cancer-related death worldwide (1). Currently, despite the tremendous advancement in the diagnosis and treatment of HCC, especially the increasing attention paid to immunotherapies targeting the tumor microenvironment (TME), only a small population of patients benefit from it owing to the therapeutic resistance and the 5-year of overall survival remains largely unsatisfactory, with the efficacy of<18% (2–4). Hence, further integrative analyses of the diversity of TME and identification of novel diagnostic and prognostic biomarkers can not only improve immunotherapeutic responsiveness but also decode the possible new molecular mechanisms of HCC initiation and progression.

This Research Topic aims to highlight the latest valuable biomarkers, gene signature sets, and prognostic-related molecular models assisting in the diagnosis, prediction of prognosis, and evaluation of immunotherapy efficacy in HCC patients. Research articles contributing to the topic are performed by multiple bioinformatic analyses underlying publicly available online databases including TCGA (http://cancergenome.nih.gov/), ICGC (https://dcc.icgc.org/), and GEO (https://www.ncbi.nlm.nih.gov/geo/), combined with *in vivo* animal models, including tumor xenograft implantation and lung metastasis assay, and *in vitro* experimental methods, such as western blot, qRT-PCR, immunochemistry, immunoprecipitation, dual-luciferase reporter gene assay, immunofluorescence, wound healing, transwell system, as well as tissue microarray (TMA).

Zheng et al. explored the biological function of decorin (DCN) secreted by cancer-related fibroblast in the progression of HCC. Mechanistically, they found that DCN inhibited the vascular invasion and metastasis of HCC by downregulating integrin β1protein expression. Rao et al. identified four hub genes of RPL19, RPL35A, RPL27A, and RPS12 by weighted gene co-expression network analysis (WGCNA) and further demonstrated that RPL19 was upregulated in HCC tissues than the adjacent liver tissues using TMA and public databases, and was intimately correlated to poor prognosis and suppressive immune response. Additionally, He et al. utilized the gene set variation analysis (GSVA) to construct the LIHC-unfavorable gene set (LUGs) and LIHC-favorable gene set (LFGs) associated with survival possibility after completely analyzing the differentially expressed genes (DEGs) in HCC datasets from TCGA, ICGC, and GEO databases. Next, they demonstrated that the patients in the high-LFG score group exerted immune activation, while the patients in the high-LUG score group were characterized by an immunosuppressive microenvironment. What is more, four genes of ESR1, EHHADH, CYB3A4, and ACADL were considered the crucial LIHC-progression characteristic genes (LCGs) and closely related to superior prognosis.

Recently, increasing evidence indicated the vital role of long non-coding RNAs (lncRNAs) in the carcinogenesis and progression of HCC (5, 6). Cao et al. analyzed the differentially expressed lncRNAs in the HCC cohort from TCGA database. They revealed that TMEM220-AS1 was low-expressed in HCC samples and TMEM220-AS1 curbs the proliferation and metastasis of HCC *via* regulating the miR484/MAGI1 axis.

Hitherto, limited knowledge is explicit concerning the prognostic value of skeletal muscle and adipose tissue mass and density in BCLC state B HCC patients with transarterial chemoembolization (TACE) treatment (7, 8). Li et al. evaluated the predictive function of skeletal muscle area (SM) and visceral adipose tissue (VAT) in this population of HCC patients and indicated that patients with VAT < 89.1 Hounsfield units (HU) experienced a prolonged survival possibility, showing the potential role of VAT in stratifying the intermediate stage HCC patients.

To elucidate the regulatory function of RNA post-transcriptional modification patterns in the malignant progression, prognosis, and TME of HCC. Li et al. constructed N6-methyladenosine (m6A) modification clusters of m6Acluster 1, m6Acluster 2, and m6Acluster 3, highly consistent with immune-inflamed, immune-desert, and immune-excluded, respectively. Moreover, they calculated the m6A scores for individual patients according to the differential m6A modification-related genes with prognostic values. The high m6A scores were involved with tumor progression, shorter survival possibility, and immunotherapy non-response. Additionally, the specific m6A regulator of YTHDF1 was overexpressed in HCC tissues and associated with low infiltration of CD3^+^ and CD8^+^ T cell types in HCC TME. Gu et al. used the HCC cohort from TCGA database to develop three 5-methylcytosine (m5C) modification subtypes and further assessed its correlation to TME, showing that Cluster-2 had a distinct survival advantage over the others. Moreover, the m5C regulator of DNMT1 was significantly upregulated in HCC samples than that in the normal tissues and was related to a poor prognosis in HCC patients. Simultaneously, upregulated expression of DNMT1 was positively correlated to several subtypes of immune cell infiltration. Xing et al. developed a prognostic model of WM-score according to the multi-layer RNA modification phenotype-related genes after integrating bioinformatic analyses of the HCC cohort in TCGA database. Later, they indicated the credible performance of WM-score value in predicting anti-tumor drug resistance and immunotherapeutic response for HCC patients.

Previous studies reported that lactate produced by aerobic glycolysis could serve as a vital signaling marker to influence the intercellular interactions, resulting in regulating the composition and function of TME. However, the specific regulatory processes are still limited (9–11). Li et al. established a lactate metabolism-related gene signature (LMRGS) using the TCGA-HCC dataset as the training cohort and the ICGC-LIRI-JP dataset was regarded as an externally validated cohort. Furthermore, they carefully evaluated the correlation of LMRGS with clinical outcomes and the TME traits of HCC patients. The results displayed that the patients within the high-LMRGS group were prone to have a shorter survival possibility and higher tumor mutation burden (TMB). Meanwhile, this population experienced a suppressed TME, with infiltrating inhibitory immune cells of follicular helper T cells and regulatory T cells and expressing repressive immune checkpoints.

This Research Topic presented the current status of updated knowledge correlated to HCC according to the comprehensive bioinformatic analyses of publicly online cancer-related databases, combined with experimental models, providing us with a variety of prognostic biomarkers or specific gene sets, as well as their predictive value of TME characteristics in HCC. We hope that this Research Topic contributes to the advancement of the diagnosis and outcome of HCC patients, especially in response to immunotherapeutic strategies.

## Author contributions

ZP specialized in the Research Topic and wrote the paper text. ZR, QX, XW, JianC, and JiangC are editors of the Research Topic. All authors contributed to the article and approved the submitted version.

## Acknowledgments

We put much gratitude to all authors of the manuscripts published on the specific Research Topic for their great endeavor and the reviewers for their stringent and exquisite review process. We still extend our gratitude to the editorial board of the Frontiers in Oncology for their great support.

## Conflict of Interest

Author JianC was employed by BioAtla, Inc.

The remaining authors declare that the research was conducted in the absence of any commercial or financial relationships that could be construed as a potential conflict of interest.

## Publisher’s note

All claims expressed in this article are solely those of the authors and do not necessarily represent those of their affiliated organizations, or those of the publisher, the editors and the reviewers. Any product that may be evaluated in this article, or claim that may be made by its manufacturer, is not guaranteed or endorsed by the publisher.
